# Guanidinoacetic Acid as a Nutritional Adjuvant to Multiple Sclerosis Therapy

**DOI:** 10.3389/fnhum.2022.871535

**Published:** 2022-05-12

**Authors:** Sergej M. Ostojic

**Affiliations:** ^1^Department of Nutrition and Public Health, University of Agder, Kristiansand, Norway; ^2^Faculty of Sport and Physical Education (FSPE) Applied Bioenergetics Lab, University of Novi Sad, Novi Sad, Serbia; ^3^Faculty of Health Sciences, University of Pécs, Pécs, Hungary

**Keywords:** guanidinoacetic acid, bioenergetics, glutamate, creatine, multiple sclerosis

## Abstract

Tackling impaired bioenergetics in multiple sclerosis (MS) has been recently recognized as an innovative approach with therapeutic potential. Guanidinoacetic acid (GAA) is an experimental nutrient that plays a significant role in high-energy phosphate metabolism. The preliminary trials suggest beneficial effects of supplemental GAA in MS, with GAA augments biomarkers of brain energy metabolism and improves patient-reported features of the disease. GAA can also impact other metabolic footprints of MS, including demyelination, oxidative stress, and GABA-glutamate imbalance. In this mini-review article, we summarize studies evaluating GAA effectiveness in MS, explore mechanisms of GAA action, and discuss the challenges of using dietary GAA as an element of MS therapy.

## Background

Multiple sclerosis (MS; ICD-11 code 8A40) is a chronic inflammatory demyelinating disease of the central nervous system with unclear causes. MS requires lifelong treatment, and many therapies are available with a substantial change in disease trajectories in the last decades (D’Amico et al., 2019; Wiendl et al., 2021), while understanding its complex etiology often provides new therapeutic targets. With a prevalence of up to 300 per 100,000 people, MS predominantly affects individuals in their early adult life, and has a considerable impact functionally, financially, and on quality of life (Thompson et al., 2018). Neuroinflammation and demyelination in MS disrupt the transmission of the signals in the parts of the nervous system, including the white matter in the optic nerve, brainstem, and spinal cord. This could result in a range of classical and unusual signs and symptoms, including a plethora of physical and mental problems (Huijbregts et al., 2006; Braley and Chervin, 2010). MS features three clinical stages: a pre-clinical stage detectable only by magnetic resonance imaging; a relapsing-remitting stage characterized by episodes of neurologic dysfunction followed by resolution; and a progressive stage, which usually evolves from the relapsing stage (Baecher-Allan et al., 2018). Besides many environmental and genetic risk factors for MS (for a detailed review, see Waubant et al., 2019), an impairment in neuronal bioenergetics has been evoked as a vital contributor to the disease (Vallée et al., 2018; Tepavcevic, 2021). Dysfunction of mitochondria, a key organelle for cell energy provision, has also been recognized in MS pathogenesis, with pathological permeability transition pore opening mediated by reactive oxygen species and calcium dysregulation might be central to mitochondrial damage and neurodegeneration in the disease (Su et al., 2013). Characterization of compounds related to mitochondrial energy metabolism in MS across body fluids (and tissues) has been suggested as a practical, easy-to-obtain laboratory tool useful to monitor MS patients and predict disease progression (Lazzarino et al., 2017). In particular, depletion of high-energy phosphates [such as adenosine triphosphate (ATP) and phosphocreatine] could accompany MS (Lazzarino et al., 2017; Adiele and Adiele, 2019), with the lower levels correlating with a more severe disability progression (Lazzarino et al., 2010). Restoring brain phosphagen bioenergetics thus emerges as a possible therapeutic approach in the disease (Ostojic, 2020), with several nutrients explored for their capacity to maintain or amplify brain energy metabolism in MS patients (Park and Choi, 2020). Guanidinoacetic acid (GAA) is an *N*-amidino derivative of glycine and an experimental nutrient that has been recently found to improve location-specific brain creatine (Ostojic, 2021a), implying its possible therapeutic value in conditions with impaired tissue bioenergetics such as MS. Besides its effects on boosting creatine levels in the human brain, GAA might also have additional metabolic roles that could benefit MS patients, including the modulation of gamma-aminobutyric acid (GABA)ergic neurotransmission and brain oxidant-antioxidant status, or a reduction of glutamate neurotoxicity. This mini-review summarizes studies evaluating GAA effectiveness in MS, discusses possible mechanisms of GAA action, and sets out open questions and future frontiers for advancing supplemental GAA as an element of MS adjuvant therapy.

## Dietary Guanidinoacetic Acid in Multiple Sclerosis

Arguably the first trial that assessed the therapeutic potential of GAA in patients with MS dates back to the early 1950s. Fallis and Lam (1952) commenced a pilot study of the effects of GAA (co-administered with betaine) in a variety of conditions of impaired neuromuscular functioning, including MS. The authors highlighted a significant energy-enhancing effect of this nutritional intervention in an entire case series, yet the article comprised data restricted to motor deficit residuals to poliomyelitis, and omitted to present findings for MS subpopulation. A seminal trial by Dr. John Aldes from the Cedars of Lebanon Hospital in Los Angeles evaluated the effects of dietary GAA plus rehabilitation in 226 MS patients over a period of 5 years (Aldes, 1957). This randomized placebo-controlled trial demonstrated favorable effects of GAA (6 g/day) together with a rehabilitation program for symptomatic relief, functional improvements, and a general sense of wellbeing in individuals with MS. In addition, patients subjected to GAA supplementation and a rehabilitation program were able to maintain normal tissue levels of phosphocreatine and ATP in the skeletal muscle after 4–12 months on this regimen. The potential of GAA to improve clinical features and tissue metabolism in MS has been confirmed in a recent case report (Ostojic et al., 2022). A middle-aged woman with secondary-progressive MS resistant to interferon beta-1alpha and corticosteroids was treated with 2 g of GAA per day (co-ingested with creatine monohydrate) for 21 days. The patient made moderate clinical progress at the follow-up, with the intensity of general fatigue, weakness, and numbness dropping from severe to mild. Magnetic resonance spectroscopy revealed increased levels of total brain creatine, choline, *N*-acetyl aspartate, and glutathione, and a drop in glutamate levels at follow-up compared to levels evaluated at initial examination. Besides MS, several recent preclinical and clinical trials demonstrated positive effects of delivering GAA to the neural tissue (McBreairty et al., 2015; Semeredi et al., 2019; Robinson et al., 2020; Ahmed-Farid et al., 2021; Seper et al., 2021; Adriano et al., 2022), corroborating its possible neurotropic potential in experimental and clinical nutrition.

## Possible Mechanisms of Guanidinoacetic Acid Action

**FIGURE 1:**
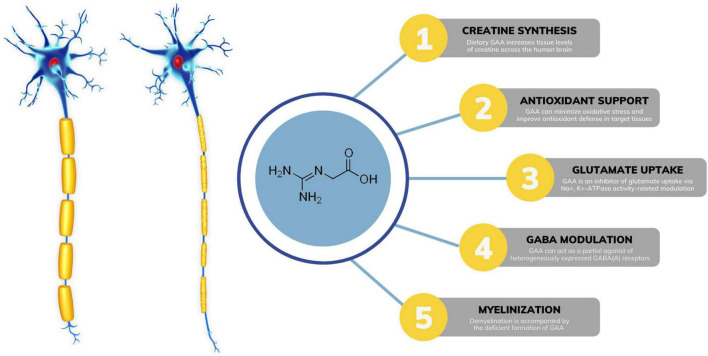
Possible mechanisms of guanidinoacetic acid (GAA) action in multiple sclerosis. GABA, gamma-aminobutyric acid; ATP, adenosine triphosphate.

GAA is a direct natural precursor of creatine. Its exogenous administration increases tissue levels of creatine across the human brain (Ostojic et al., 2017), which may tackle impaired creatine bioenergetics seen in MS. Preclinical trials suggest that creatine can act as a neuroprotective agent by increasing ATP production and enhancing oligodendrocyte survival after demyelinating injury, including MS (Chamberlain et al., 2017). Interestingly, supplemental GAA might be even better to affect cerebral creatine concentrations than creatine itself, perhaps due to more favorable transport kinetics throughout the blood-brain barrier (Ostojic et al., 2016). Besides augmenting creatine levels, GAA might affect other neuromodulating compounds in MS (Figure 1). Demyelination is often characterized by various neurochemical abnormalities in GABA-glutamate metabolism (Swanberg et al., 2019), including dysfunctional glutamatergic excitation and GABAergic inhibition. GAA can reverse irregularities in glutamate-GABA turnover linked to MS, acting as an inhibitor of glutamate uptake *via* Na^+^, K^+^ -ATPase activity-related modulation (Zugno et al., 2007; Marques et al., 2019). A GAA-driven reduction in brain glutamate levels has been corroborated in human studies. A strong trend has been reported for reduced glutamate in white matter after 8 weeks of GAA supplementation (decrease of ∼4.5% from baseline levels) in healthy men (Ostojic and Ostojic, 2018). The lowering of glutamate levels after GAA administration (∼9.5%) was found in the gyrus cinguli of a patient suffering from acute secondary progressive MS (Ostojic et al., 2022). In addition, GAA can interact with neuronal GABA receptors, implying its possible role in GABA release and utilization in the central nervous system (Cupello et al., 2008; Chebib et al., 2009; Schulze et al., 2016). Specifically, GAA can act as a partial agonist of heterogeneously expressed GABA_*A*_ receptors (Neu et al., 2002); this might counteract GABAergic inhibition seen in MS and potentially be of therapeutic value. Interestingly, creatine has no effect as a GABA agonist, antagonist, or modulator (Chebib et al., 2009).

Accumulating evidence indicates that oxidative stress plays a major role in the pathogenesis of MS (Ohl et al., 2016). Preclinical trials suggest that dietary GAA can minimize oxidative stress and improve antioxidant defense in target tissues (Aziza et al., 2020; Oviedo-Rondón and Córdova-Noboa, 2020; Zhao et al., 2021), possibly *via* mechanisms related to attenuating lipid peroxidation. A human study demonstrated augmented brain glutathione levels, an antioxidant indispensable for preventing lipid peroxidation in brain cells, after a 21-day GAA intervention in MS (Ostojic et al., 2022). Finally, secondary findings from an interesting case report suggest that GAA might be associated with myelinogenesis in a demyelinating disease similar to MS (Brunetti-Pierri et al., 2008). The authors detected possible irregularities in brain GAA levels in a patient with GM1 gangliosidosis and diffuse reduction of myelination, accompanied by redundant and inappropriately folded myelin. Gyrate atrophy of the choroid and retina is another disease with demyelination, and it appears that the disease is accompanied by the deficient formation of GAA (Sipilä et al., 1980). Although of uncertain etiology and significance, GAA alterations in demyelination might be attributed to disturbed axon-oligodendrocytes interactions.

## Open Questions for Guanidinoacetic Acid Use in Multiple Sclerosis

Although preliminary trials demonstrated favorable results of using GAA as an adjunct component of MS treatment, many issues remain to be addressed before its advancement to everyday care. First of all, we still lack well-sampled longitudinal pharmacovigilance studies with GAA in MS settings. Addressing GAA safety is of utmost importance keeping in mind that animal and *in vitro* studies suggest possible neurotoxicity of GAA when accumulated in supraphysiological doses (for a detailed review, see Ostojic, 2021b). The possibility of dietary GAA accruing in the human brain is highly unlikely (Ostojic and Ostojic, 2018). Still, its use in MS might require careful titration in aim to adjust the dose for the maximum benefit without adverse effects. Until now, a daily dosage of GAA administered to patients with MS was up to 75 mg per kilogram of body weight, with most studies using ∼25 mg of GAA per kg of body weight. Another open question includes the magnitude of exogenous GAA uptake from the circulation into the MS-compromised brain. GAA has a finite capacity to cross the blood-brain barrier (BBB) under physiological conditions (Tachikawa et al., 2009), and an MS-driven disruption of the BBB might affect net GAA uptake. For instance, an early event in MS is a diminished function of the BBB (Kamphuis et al., 2015) which could facilitate transporting GAA into the brain; this perhaps requires an additional adjustment of GAA dosage used in MS. Furthermore, GAA appears to be effective in MS when co-administered with other nutrients and/or therapeutic exercise; no clinical trials have evaluated the effects of sole GAA in the disease.

## Conclusion

MS is a complex, debilitating disease. Disease trajectories have been substantially changed by the approval of several disease-modifying therapies, and research is now moving also on nutraceuticals. Few preliminary clinical trials suggest that dietary GAA might be fairly beneficial in improving patient- and clinician-reported outcomes when added as a nutritional component to the MS treatment protocol. This likely happens due to a GAA-driven modulation of brain metabolism involving creatine bioenergetics and neurotransmitters turnover. Those promising findings call for long-term randomized controlled trials with GAA across MS cohorts.

## Author Contributions

SO designed, wrote the manuscript, revised, approved the final version of the manuscript, and has primary responsibility for the final content.

## Conflict of Interest

SO serves as a member of the Scientific Advisory Board on creatine in health and medicine (AlzChem LLC). SO owns patent “Sports Supplements Based on Liquid Creatine” at European Patent Office (WO2019150323 A1), and active patent application “Synergistic Creatine” at United Kingdom Intellectual Property Office (GB2012773.4). SO has served as a speaker at Abbott Nutrition, a consultant of Allied Beverages Adriatic and IMLEK, and has received research funding related to creatine from the Serbian Ministry of Education, Science, and Technological Development, Provincial Secretariat for Higher Education and Scientific Research, AlzChem GmbH, KW Pfannenschmidt GmbH, ThermoLife International LLC, and Hueston Hennigan LLP. SO does not own stocks and shares in any organization.

## Publisher’s Note

All claims expressed in this article are solely those of the authors and do not necessarily represent those of their affiliated organizations, or those of the publisher, the editors and the reviewers. Any product that may be evaluated in this article, or claim that may be made by its manufacturer, is not guaranteed or endorsed by the publisher.
